# Clinical Characteristics of Hospitalized Pediatric Patients With Hypertensive Crisis—A Retrospective, Single-Center Study in China

**DOI:** 10.3389/fcvm.2022.891804

**Published:** 2022-05-31

**Authors:** Hongjun Ba, Huimin Peng, Lingling Xu, Youzhen Qin, Huisheng Wang

**Affiliations:** ^1^Department of Paediatric Cardiology, Heart Centre, The First Affiliated Hospital, Sun Yat-sen University, Guangzhou, China; ^2^Key Laboratory on Assisted Circulation, Ministry of Health, Guangzhou, China; ^3^Department of Pediatrics, The First Affiliated Hospital of Sun Yat-sen University, Guangzhou, China

**Keywords:** hypertensive crisis, hypertensive emergency, hypertension, hypertensive urgency, children

## Abstract

**Background:**

A hypertensive crisis is a medical emergency that causes acute damage to multiple organs. However, the etiology, clinical features, and prognosis of hypertensive crisis in Chinese children remain relatively unknown. The purpose of this study was to analyze the clinical characteristics of pediatric hypertensive crisis patients from a single center in China.

**Methods:**

We analyzed data from 70 children with hypertensive crisis between January, 2000, and January, 2022. The patients were divided into two groups: those diagnosed with a hypertensive emergency (*n* = 46) and those diagnosed with hypertensive urgency (*n* = 24). Baseline etiologies and risk factors were compared between the two groups. The following data were collected and analyzed: age, sex, weight, height, family history of hypertension, blood pressure, clinical manifestations of hypertensive crisis, underlying causes, biochemical indicators, and antihypertensive drugs.

**Results:**

The major symptoms of hypertensive crisis were headache (*n* = 31, 44.29%), followed by visual symptoms (*n* = 15, 21.43%), and dizziness (*n* = 13, 18.57%). Further analysis showed that the incidence of convulsions was significantly higher in patients with hypertensive emergency than those with hypertensive urgency (χ^2^ = 5.38, *p* = 0.02). The leading underlying causes were renal disease (*n* = 34, 48.57%), followed by vascular disease (*n* = 11, 15.71%), essential hypertension (*n* = 9, 12.86%), oncological disease (*n* = 9, 12.86%), central nervous system disease (*n* = 3, 4.29%), endocrine and metabolic diseases (*n* = 2, 2.86%), and other (one case with lead poisoning, one case with histiocytosis). End-organ damage occurred in 46 patients with hypertensive crisis, including retinal damage (*n* = 20, 43.48%), brain damage (*n* = 19, 41.30%), heart damage (*n* = 15, 32.61%), and renal damage (*n* = 3, 6.52%). Hypertensive crisis was most common among children aged 7–12 years. Among children aged 13–18 years, hypertensive urgency was more common than hypertensive emergency. The incidence of dyslipidemia, elevated serum creatinine, and elevated uric acid did not differ significantly between the two groups. Most patients with hypertensive crisis need combined antihypertensive therapy (*n* = 60, 85.71%). There were no cases of mortality.

**Conclusions:**

Hypertensive crisis is caused by secondary diseases, especially renal disease and vascular disease, in the majority of pediatric patients. Combination therapy with antihypertensive agents and treatment of secondary etiology results in a good prognosis.

## Introduction

Hypertension (HTN) in children has become a common health problem. Hypertensive crisis is a potentially life-threatening cardiovascular emergency (1, 2). Compared with adults, pediatric HTN is mostly asymptomatic with most children having no history of HTN (2). In addition, since accurate measurement of pediatric blood pressure (BP) is difficult, hypertensive crises in children are often misdiagnosed and missed. Hypertensive crisis is categorized as hypertensive urgency and hypertensive emergency; the latter is associated with end-organ damage (heart, brain, kidneys, and arteries) and is a potentially life-threatening condition (3, 4). Due to the lack of a unified definition, estimating the incidence of hypertensive crises in children and adolescents is difficult. A single-center study in London, UK reported that 24% of patients receiving treatment for HTN had severe HTN, which was defined as BP exceeding that of the 99th percentile of the population (5). A cross-sectional study on HTN among middle school students in Houston, USA, revealed that the incidence of grade 2 HTN was 0.6% (6). Moreover, based on limited data, 12–14% of patients with hypertensive crisis were found to be <1 year of age (5). A study in Taiwan found that 78% of children with hypertensive crisis were younger than seven years, while 44% were younger than 13 years of age (4). Studies have observed a sex difference, with a significantly higher incidence rate in boys than girls (4).

Hypertensive crisis in children and adolescents can occur due to HTN caused by any etiology; however, it is most commonly due to secondary HTN. The etiologies differ as per the age groups. The etiology of neonatal hypertension mainly includes coarctation of the aorta, renal artery or venous thrombosis, and polycystic kidney disease (7), whereas essential hypertension, renal diseases, endocrine diseases, autoimmune diseases, and medications are important etiologies in older children and adolescents (8, 9). The objective of this study was to analyze the clinical features, etiology, and treatment of children with hypertensive crisis.

## Methods

### Patient Population

We conducted a retrospective chart review of all patients aged 18 years and younger with a diagnosis of HTN from January 2000 to January 2022 in our pediatric ward at the First Affiliated Hospital of Sun Yat-sen University, a national regional medical center in China. The exclusion criteria were as follows: BP below the 95th percentile, a final diagnosis of transient HTN, incomplete data, and no repeated BP measurements. The diagnostic criterion for childhood hypertensive crisis is BP over the 99th percentile for children of the same sex, age, and height plus 5 mmHg. Patients aged 3–17 years refer to the Chinese guidelines (10), and those younger than 3 years refer to the US guidelines (9). Patients with hypertensive crisis were further subdivided into two severity groups: hypertensive urgency and hypertensive emergency, based on the presence of end-organ damage.

Hypertensive emergency was defined as HTN in the presence of acute or ongoing end-organ damage or HTN associated with an immediate life-threatening event requiring immediate intervention to reduce BP (11, 12). Hypertensive urgency was defined as systolic blood pressure (SBP) or diastolic blood pressure (DBP) higher than the 99th percentile plus 5 mmHg accompanied by any complication related to HTN without evidence of end-organ damage (12). End-organ damage was defined as impaired renal, myocardial, hepatic, or hematologic functioning or neurological manifestations due to HTN.

A total of 476 patients presented to our pediatric ward with a diagnosis of primary or secondary HTN. Six patients were excluded due to a final diagnosis of transient HTN, and eight were excluded due to inadequate data. Finally, a total of 462 hypertensive patients with complete data were enrolled, and a total of 70 patients were diagnosed with hypertensive crisis. This study was approved by the Human Subjects Review Committee of the hospital. Informed consent was waived due to the retrospective nature of this study. The patients were divided into three age groups: preschool age (< or equal to 6 years of age), elementary school age (7–12 years of age), and adolescents (13–18 years of age).

### Study Variables

The following data were collected and analyzed: age, sex, weight, height, family history of HTN, BP, clinical manifestations of hypertensive crisis (dizziness, headache, nausea/vomiting, visual symptoms, seizure/type, altered consciousness, chest pain, end-organ damage), anti-hypertensive drugs, biochemical indicators (blood lipids, serum creatinine, serum uric acid), and underlying causes (renal disease, cardiovascular disease, essential HTN, central nervous system (CNS) factors, endocrine/metabolic disorders, and oncological disease). Central nervous system factors refer to CNS abnormalities that cause HTN, not including hypertensive encephalopathy which occurs secondary to HTN. Essential HTN was diagnosed after excluding secondary causes of HTN through various investigations including electrocardiography, a metabolic panel, renal function tests, hemoglobin and routine urine tests, echocardiography, renal ultrasound, plasma renin activity, plasma aldosterone, thyroid-stimulating hormone, and 24-h urine free cortisol levels. Dyslipidemia was determined by reference (13).

The BP levels, etiology, severity, and clinical manifestations were compared among children by age group and compared between patients with hypertensive emergency and hypertensive urgency.

Height z-scores and weight z-scores were calculated by referring to the growth and development data of normal children in China (14).

Hypertensive encephalopathy was defined as BP > 150–160/100–110 mmHg with more than one neurological symptom out of the following: vomiting, diplopia, severe headache, transient blindness, convulsions, or loss of consciousness (15).

Retinal damage in hypertensive crisis was defined as hypertensive crisis with retinopathy (16).

Hypertensive crisis with renal damage was defined as hypertension with nocturia, mild proteinuria, or elevated serum creatinine, excluding primary nephropathy with hypertension (1).

Hypertensive crisis with heart damage was defined as markedly elevated blood pressure associated with acute heart failure in the absence of other causes (17).

### Statistical Analysis

All statistical analyses were performed using the chi-square test and the Kruskal Wallis test. The results of the descriptive analyses of the independent variables are reported as percentages and mean ± SD. Statistical significance was set at *p* < 0.05. Statistical analyses were performed using SPSS software (version 17.0; SPSS Inc., Chicago, IL, United States).

## Results

### Participant Characteristics

**Figure 1:**
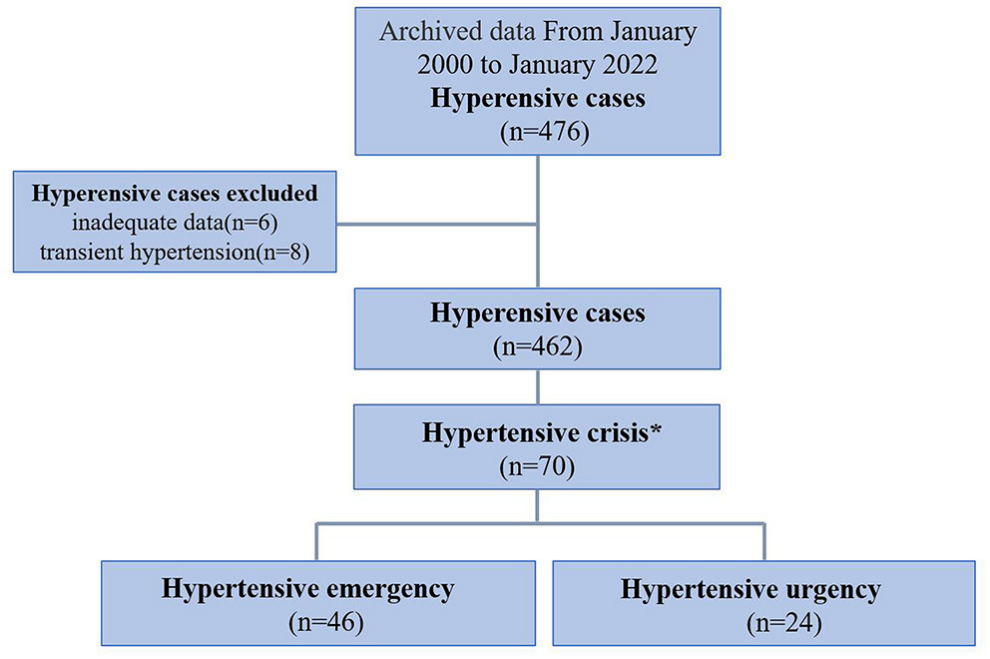
Flow chart showing the process of patient selection. A total of 476 children were diagnosed with hypertension from January 2000 to January 2022. Excluding 6 patients with incomplete data and 8 patients with transient hypertension, 462 patients met the inclusion criteria. Among them, 70 patients met the criteria of hypertensive crisis and were then divided into hypertensive emergency group (with acute or ongoing target organ lesions) and hypertensive urgency group (without evidence of target organ lesions). *Hypertensive crisis was defined as BP ≥ 99th percentile + 5 mmHg.

From January 2000 to January 2022, a total of 462 HTN patients with complete data were assessed, and a total of 70 patients met the criteria for hypertensive crisis (Figure 1), including 24 cases (34.29%) with hypertensive urgency and 46 cases (65.71%) with hypertensive emergency (incidence ratio 1:1.9). The male-to-female incidence ratio was 2:1 (boys, *n* = 47; girls, *n* = 23). Most patients were in the school-age group (*n* = 50, 71.43%). A Family history of HTN was noted in patients (*n* = 5, 7.14%). The SBP and DBP were significantly elevated in all (*n* = 70, 100.00%) and most (*n* = 63, 90.00%) pediatric patients with hypertensive crisis, respectively (Table 1).

**Table 1:** Characteristics of the patients with hypertensive crisis by age group.

	**Age(years)**			
**Variables**	**≤6 (*n* = 20)**	**7–12 (*n* = 38)**	**13–18 (*n* = 12)**	***p*-value**
Gender				
Female	7 (35.00)	12 (31.58)	4 (33.33)	0.965
Male	13 (65.00)	26 (68.42)	8 (63.67)	
Height (cm)	99.11 ± 16.76	133.00 ± 12.25	152.62 ± 19.22	<0.001
Height z-score (SD)	−0.31 ± 1.84	−0.56 ± 1.80	−1.25 ± 2.65	0.772
Weight (kg)	16.65 ± 7.41	29.47 ± 7.56	45.31 ± 17.58	<0.001
Weight z-score (SD)	−0.46 ± 1.86	0.61 ± 1.12	−1.05 ± 2.08	0.874
Family history	0 (0.00)	3 (7.89)	2 (16.67)	0.201
Blood pressure				
SBP > 99th percentile+5	20 (100.00)	38 (100.00)	12 (100.00)	–
DBP > 99th percentile+5	16 (80.00)	38 (100.00)	9 (75.00)	0.009
Clinical presentations				
Headache	1 (5.00)	20 (52.63)	10 (83.33)	<0.001
Vomiting	2 (10.00)	6 (15.79)	3 (25.00)	0.529
Visual symptoms	3 (15.00)	12 (31.58)	0 (0.00)	0.048
Convulsions	3 (15.00)	6 (15.79)	0 (0.00)	0.342
Dizziness	2 (10.00)	10 (26.32)	1 (8.33)	0.191
Chest pain	0 (0.00)	1 (2.63)	1 (8.33)	0.388
Conscious disturbance	2 (10.00)	3 (7.89)	0 (0.00)	0.548
End-organ damage	13 (65.00)	26 (68.42)	7 (58.33)	0.811

*Gender, family history, blood pressure, and clinical presentations were described as number (percentage). Height, height z-score, weight, and weight z-score was described as mean ± standard deviation. SD, standard deviation*.

### Etiology of Hypertensive Crisis

The leading underlying causes of hypertensive crisis were renal disease (*n* = 34, 48.57%), followed by vascular disease (*n* = 11, 15.71%), essential HTN (*n* = 9, 12.86%), oncological disease (*n* = 9, 12.86%), CNS disease (*n* = 3, 4.29%), endocrine and metabolic diseases (*n* = 2, 2.86%), and other (one case with lead poisoning, one case with histiocytosis) (Table 2). Renal diseases include renal artery stenosis (*n* = 15, 44.12%) and renal parenchymal disease (*n* = 19, 55.88%). Renal parenchymal diseases included end-stage renal disease (*n* = 10, 52.63%), acute nephritis (*n* = 3, 15.79%), immunoglobulin A nephropathy (*n* = 2, 10.53%), lupus nephritis (*n* = 2, 10.53%), congenital renal dysplasia (*n* = 1, 5.26%), and duplex kidney (*n* = 1, 5.26%). Vascular diseases included coarctation of the aorta (*n* = 4, 36.36%) and Takayasu's arteritis (*n* = 7, 63.64%). Oncological diseases included pheochromocytoma (*n* = 5, 55.56%), nephroblastoma (*n* = 2, 22.22%), neuroblastoma (n = 1, 11.11%), and adrenal cortical carcinoma (*n* = 1, 11.11%). Endocrine and metabolic diseases included adrenal hyperplasia (*n* = 1, 50.00%) and obesity (*n* = 1, 50.00%). CNS diseases included traumatic cerebral hemorrhage (*n* = 1, 33.33%), Guillain-Barre syndrome (*n* = 1, 33.33%), and retrobulbar neuritis (*n* = 1, 33.33%).

**Table 2:** Etiology of the patients with hypertensive crisis.

**Etiology**	**Hypertensive emergencies (*****n*** **=** **46)**		**Hypertensive urgencies (*****n*** **=** **24)**		***p* value**
	***N***	**percentage**	***N***	**percentage**	
Vascular disease	7	15.21	4	16.67	0.874
Coarctation of aorta	1	2.17	3	12.50	0.077
Takayasu's arteritis	6	13.04	1	4.17	0.240
Renal disease	25	54.35	9	37.50	0.181
Renal parenchyma disease	13	28.26	6	25.00	0.771
Renal artery stenosis	12	26.09	3	12.50	0.189
Oncology	8	17.39	1	4.17	0.117
CNS	2	4.35	1	4.17	0.972
Essential HTN	2	4.35	7	29.17	0.003
Endocrine/metabolic	1	2.17	1	4.17	0.635
Other	1	2.17	1	4.17	0.635

*CNS, central nervous system; HTN, hypertension*.

The top three causes of hypertensive emergency were renal disease (*n* = 25, 54.35%), oncological diseases (*n* = 8, 17.39%), and vascular diseases (*n* = 7, 15.22%). The top three causes of hypertensive urgency were renal disease (*n* = 9, 37.50%), essential HTN (*n* = 7, 29.17%), and vascular diseases (n = 4, 16.67%). In addition, some rare etiologies were found: one case in the hypertensive emergency group was caused by lead poisoning, and one case in the hypertensive urgency was due to histiocytosis.

Renal disease was the main cause of hypertensive crisis in both emergency and urgency groups. Compared with hypertensive emergency patients, the incidence rate of essential HTN was higher in those with hypertensive urgency (χ^2^ = 8.67, *p* = 0.003). There were no significant differences in the incidence rates of renal diseases, tumors, endocrine and metabolic diseases, and CNS diseases between the hypertensive emergency and hypertensive urgency patients (Table 2). There was no also significant difference in the incidence of dyslipidemia, elevated serum creatinine and uric acid between the two groups (Table 3).

**Table 3:** Variables of hypertensive emergencies and urgencies.

**Variables**	**Hypertensive emergencies (*n*, percentage)**	**Hypertensive urgencies (*n*, percentage)**	***p*-value**
Sex, male	29 (63.04)	18 (75.00)	0.312
Elevated TG	16 (34.78)	5 (20.83)	0.227
Elevated TC	19 (41.30)	6 (25.00)	0.177
Elevated LDL-c	17 (36.96)	5 (20.83)	0.168
Reduced HDL-c	8 (17.39)	2 (8.33)	0.504
Elevated SCr	17 (36.96)	6 (25.00)	0.312
Elevated SUc	11 (23.91)	6 (25.00)	0.920
Albuminuria	14 (30.43)	6 (25.00)	0.633

*TG, triglycerides; TC, total cholesterol; LDL-c, low-density lipoprotein cholesterol; HDL-c, high-density lipoprotein cholesterol; SCr, serum creatinine; SUc, serum uric acid*.

### Symptomatology and Manifestations of End-Organ Damage

**Figure 2:**
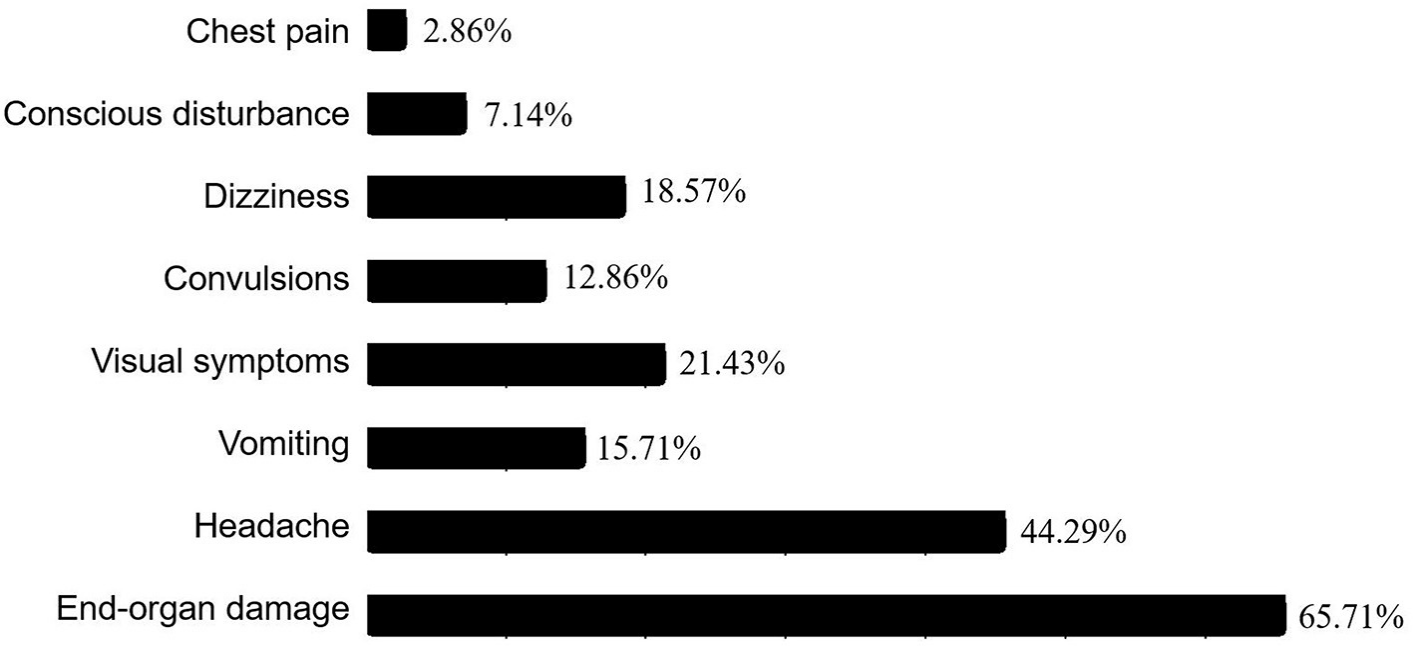
Ratios of clinical manifestations in the pediatric patients with hypertensive crisis. Among the 70 patients with hypertensive crisis, chest pain was present in 2 cases (2.86%), conscious disturbance in 5 cases (7.14%), dizziness in 13 cases (18.57%), convulsions in 9 cases (12.86%), visual symptoms in 15 cases (21.43%), vomiting in 11 cases (15.71%), headache in 31 cases (44.29%), and end-organ damage in 46 cases (65.71%).

**Figure 3:**
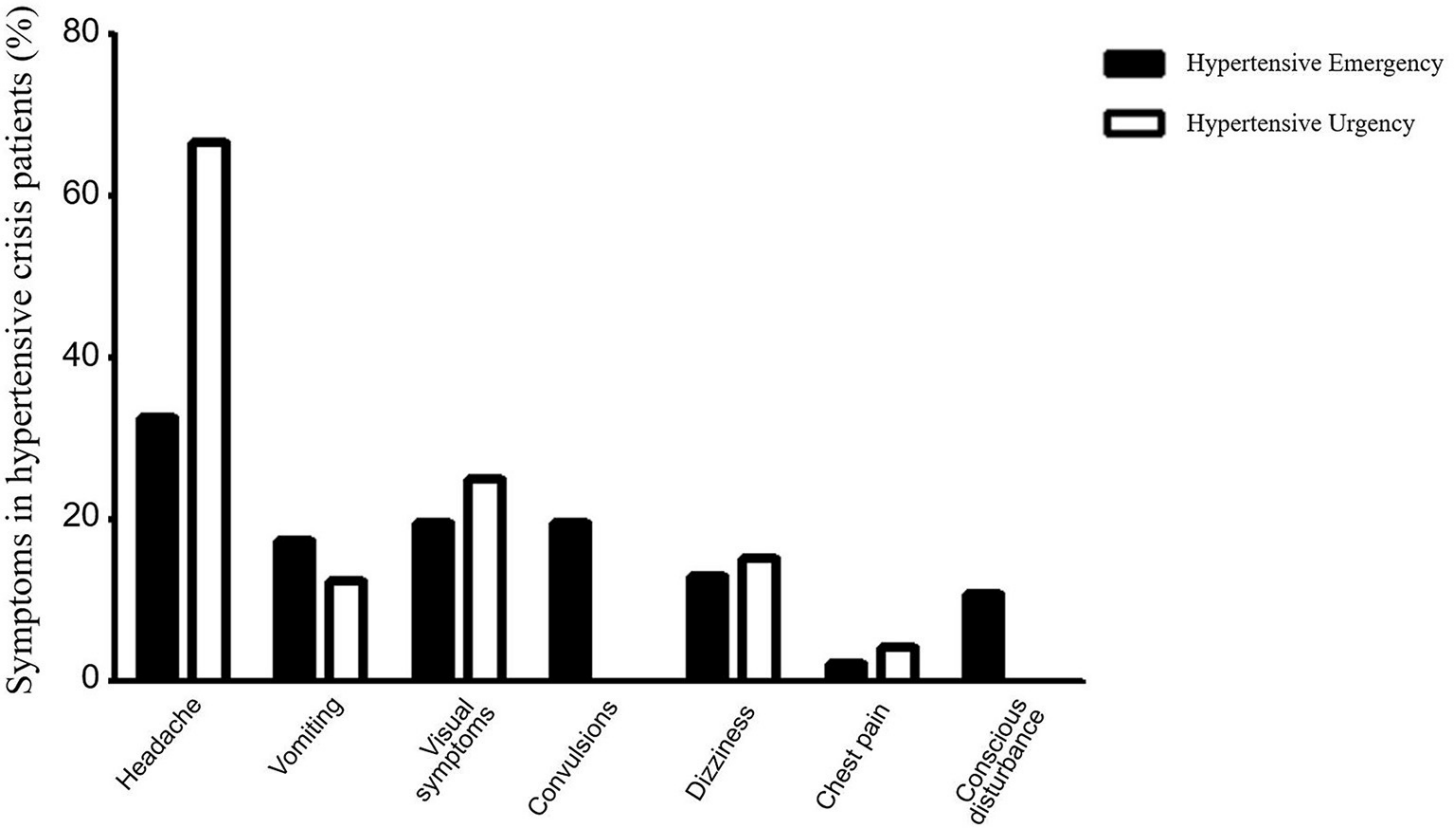
Comparison of clinical symptoms between the hypertensive emergency group and the hypertensive urgency group. The solid columns represent the hypertensive emergency group and the hollow columns represent the hypertensive urgency group. In the hypertensive emergency group, headache was present in 15 cases (32.61%), vomiting in 8 cases (17.39%), vision symptoms in 9 cases (19.57%), convulsions in 9 cases (19.57%), dizziness in 6 cases (13.04%), chest pain in 1 case (2.17%), and conscious disturbance in 5 cases (10.87%). In the hypertensive urgency group, headache was present in 16 cases (66.67%), vomiting in 3 cases (12.50%), visual symptoms in 6 cases (25.00%), dizziness in 7 cases (29.17%), and chest pain in 1 case (4.17%), with no cases of convulsion or disturbance of consciousness. Further analysis showed that the incidence of convulsions was significantly higher in patients with hypertensive emergency than those with hypertensive urgency (χ^2^= 5.38, *p* = 0.02). The incidence of headache was significantly higher in patients with hypertensive urgency than those with hypertensive emergency (χ^2^= 7.41, *p* = 0.006).

The major symptoms of hypertensive crisis were headache (*n* = 31, 44.29%), followed by visual symptoms (*n* = 15, 21.43%), and dizziness (*n* = 13, 18.57%) (Figure 2). Further subgroup analysis showed that the incidence of convulsions was significantly higher in patients with hypertensive emergency than those with hypertensive urgency (χ^2^= 5.38, *p* = 0.02). And the incidence of headache was significantly higher in patients with hypertensive urgency than those with hypertensive emergency (χ^2^= 7.41, *p* = 0.006). There were no significant differences in the incidence of headache, vomiting, visual symptoms, dizziness, chest pain, and conscious disturbance between the two groups (Figure 3).

**Figure 4:**
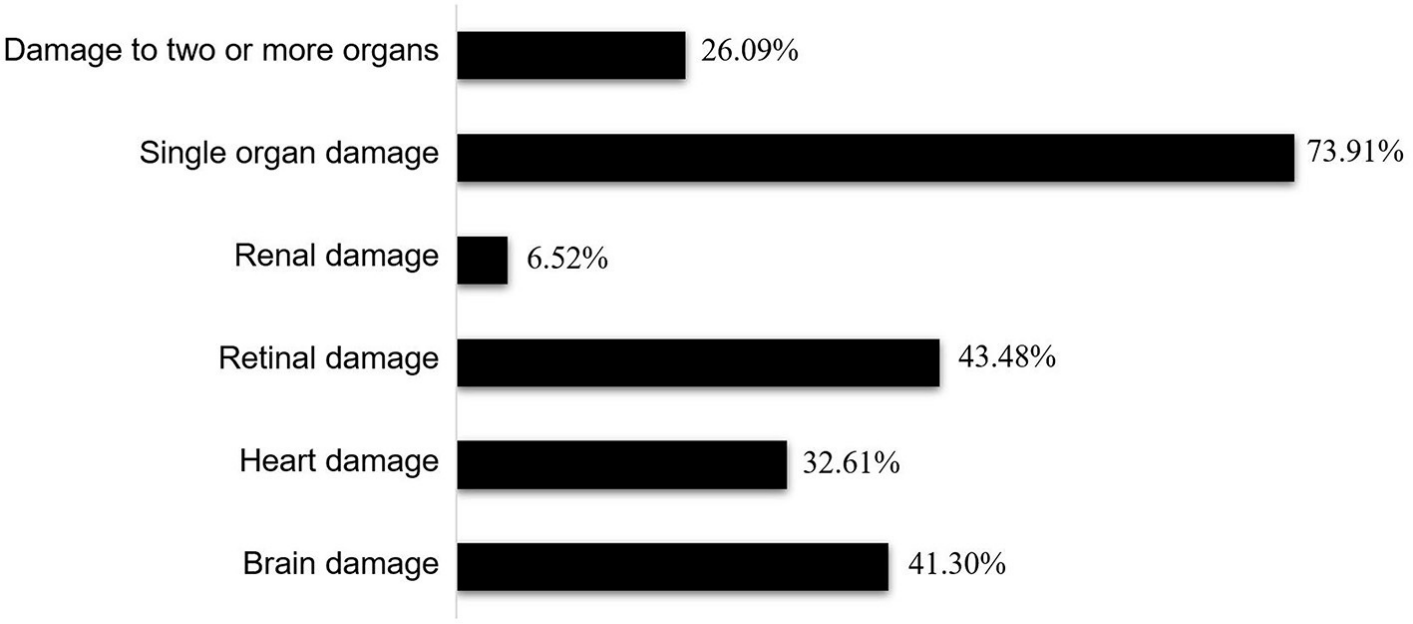
Ratios of end-organ damage in the hypertensive emergency group. Among the 46 patients with hypertensive emergency, 12 (26.09%) had damage to two or more organs while 34 (73.91%) had damage to one organ. Damages to the retina (20 cases, 43.48%) and brain (19 cases, 41.30%) were most frequent, followed by damage to the heart (15 cases, 32.61%), with the kidney (3 cases, 6.52%) being the least affected.

End-organ damage occurred in 46 patients with a hypertensive crisis and included retinal damage (*n* = 20, 43.48%), brain damage (*n* = 19, 41.30%), heart damage (*n* = 15, 32.61%), and renal damage (*n* = 3, 6.52%); retinal damage and brain damage were the most common conditions. The end-organ damage found in these patients was mainly limited to a single organ (*n* = 34, 73.91%), and the occurrence of multiple organ damage (*n* = 12, 26.09%) was rare (Figure 4). Retinal damage manifested as blurred vision, decreased vision, and diplopia. Headaches, dizziness, and convulsions were the main manifestations of brain damage, while cardiac failure was the main manifestation of heart damage. The main clinical manifestations of patients with heart failure included tachypnea (*n* = 14, 93.33%), tachycardia (*n* = 15, 100%), profuse sweating (*n* = 8, 53.33%), and hepatomegaly (*n* = 11, 73.33%). Chest X-rays revealed cardiomegaly (*n* = 13, 86.67%), and echocardiography revealed ventricular dilation (*n* = 15, 100%) and a reduced left ventricular ejection fraction (*n* = 8, 53.33%), while electrocardiography revealed sinus tachycardia (*n* = 15, 100%). In addition, all patients had elevated N-terminal prohormone of brain natriuretic peptide (NT-proBNP) levels. Hypertensive encephalopathy was the main manifestation of brain damage in hypertensive crisis. Magnetic resonance imaging (MRI) of brain revealed increased signal intensity in the subcortical white matter and cortical gray matter of the parieto-occipital area, cerebellum, and basal ganglia.

The average time for heart failure patients to return to normal heart function was 20.06 ± 0.03 days. The cardiothoracic ratio decreased from 0.59 ± 0.03 to 0.48 ± 0.02. The left ventricular ejection fraction increased from 0.52 ± 0.05 to 0.58 ± 0.02. The urine volume increased from 0.65 ± 0.12 ml/kg/h to 2.1 ± 0.29 ml/kg/h, and NT-proBNP decreased from 4178.0 ± 860.0 pg/ml to 182.33 ± 36.81 pg/ml. Urine volume, urine routine, and serum creatinine recovered in three patients with renal damage. Patients with retinal damage take an average of 6 months to return to normal. The mean time for brain MRI to return to normal in patients with brain injury is 8 months.

In patients with heart and renal damage, all patients recovered. Retinal damage was relieved in 17 cases (85%) and persisted in 3 cases (15%). 16 cases (84.21%) with brain damage were recovered, and three cases (15.79%) were not recovered. Those who did not recover also showed significant improvement.

### Hypertensive Crisis by Age Group and Treatment

**Figure 5:**
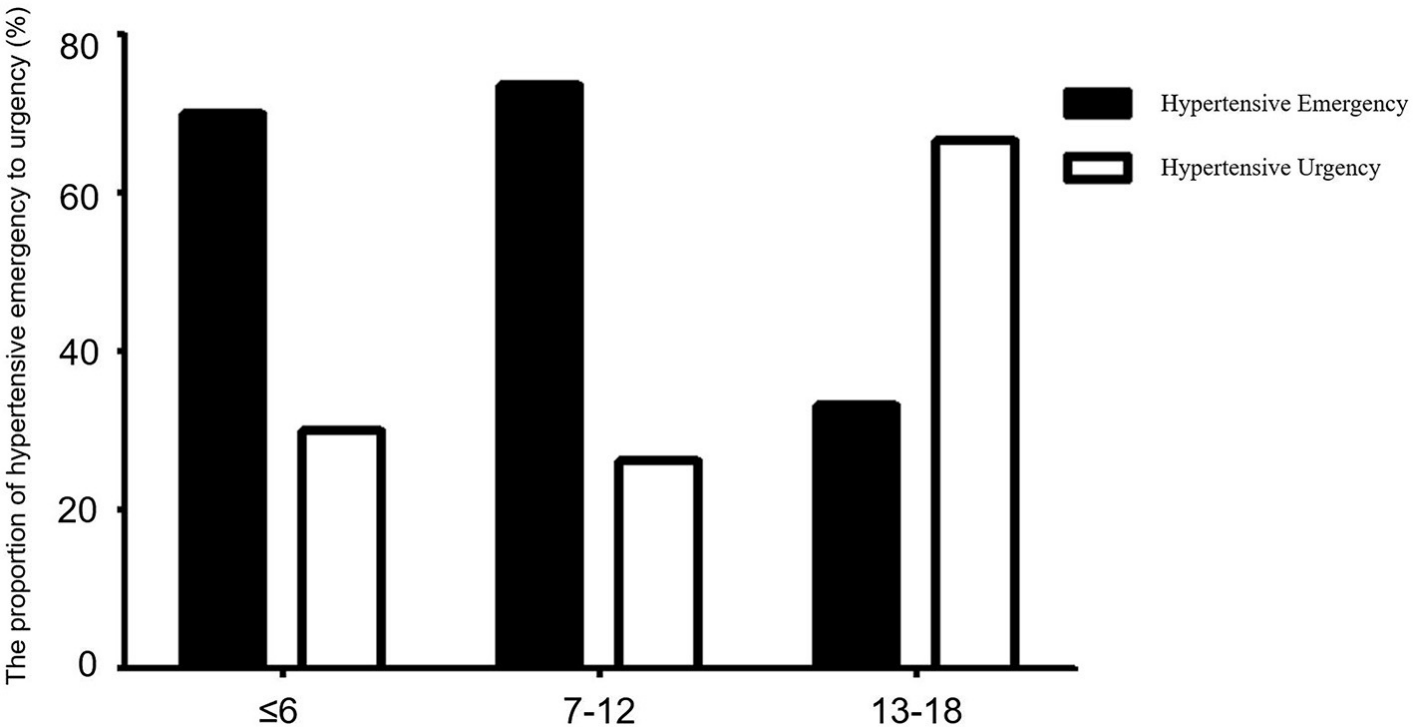
Comparison of the proportions of hypertensive emergency and hypertensive urgency between different age groups. Solid and hollow columns represent hypertensive emergency and hypertensive urgency patients, respectively. There were 20 cases aged ≤ 6 years, of whom 14 (70.00%) were hypertensive emergency and 6 (30.00%) were hypertensive urgency. There were 38 patients aged 7–12 years, of whom 28 (73.68%) were hypertensive emergency, 10 (26.32%) were hypertensive urgency. There were 12 patients aged 13–18 years, of whom 4, (33.33%) were hypertensive emergency, 8 (66.67%) were hypertensive urgency. Hypertensive emergency was more common than hypertensive urgency among children aged 7–12 years (χ^2^ = 17.05, *p* < 0.001).

We compared the number of hypertensive crises in different age groups; they were most common among children aged 7–12 years, while the number of hypertensive crises in patients under 1 year of age was minimal. Hypertensive emergency was more common than hypertensive urgency among children aged 7–12 years (χ^2^ = 17.05, *p* < 0.001) (Figure 5).

In hypertensive emergency group, a single antihypertensive was used in two cases, both in essential hypertension patients. The used drugs were metoprolol and nifedipine. Forty-four patients were treated with a combination of antihypertensive drugs, 16 of which were treated with a combination of two drugs. The most common etiologies included takayasu's arteritis, non-pheochromocytoma tumors, and renal arterial stenosis. The commonly used drug regimen was metoprolol + hydrochlorothiazide in 10 cases, metoprolol + nifedipine in four cases, and captopril + nifedipine in two cases. 28 cases were treated with three-drug combination. Patients requiring the combination of the three drugs mainly presented with pheochromocytoma, severe renal artery stenosis, and renal failure. The commonly used medication regimen was metoprolol + nifedipine + hydrochlorothiazide in 10 cases, enalapril + nifedipine + hydrochlorothiazide in 8 cases, and metoprolol + terazosin + nifedipine in five cases. A total of 17 patients received intravenous antihypertensive drugs. Nitroprusside (*n* = 10, 58.82%) and nicardipine (*n* = 5, 29.41%) were the most commonly used intravenous antihypertensive drugs, followed by urapidil (*n* = 2, 11.76%). The time to transition from intravenous to oral treatment was 30.40 ± 3.20 h in the nitroprusside and 31.80 ± 3.35 h in the nicardipine group.

In hypertensive urgency group, a single antihypertensive was used in eight cases, mainly in essential hypertension patients. The most commonly used drugs were metoprolol in two cases, enalapril in two cases, captopril in three cases, and nifedipine in one case. 16 patients received combined antihypertensives. In these patients, 12 patients were treated with a combination of two antihypertensives. The most common etiologies included Takayasu's arteritis and renal stenosis. The commonly used drug regimen was metoprolol + hydrochlorothiazide in two cases, metoprolol + nifedipine in four cases, and captopril + nifedipine in three cases. Four cases were treated with three-drug combination. Patients requiring the combination of the three drugs mainly presented with renal failure. The commonly used medication regimen was metoprolol + nifedipine + hydrochlorothiazide in two cases and enalapril + nifedipine + hydrochlorothiazide in two cases. In addition to antihypertensive therapy, treatment of secondary etiology was also carried out, such as renal transplantation in cases of end-stage renal disease, interventional treatment of renal artery stenosis, and surgical treatment of oncological disease. There were no cases of mortality.

## Discussion

Compared with adults, relatively few pediatric patients with hypertensive crisis have been reported. Moreover, the diagnosis of hypertensive crisis in children is often difficult due to the lack of typical clinical manifestations (3). In our study, a total of 462 hypertensive patients with complete data were collected, of which 70 (15.15%) met the criteria for hypertensive crisis. The epidemiology of hypertensive crisis in children is uncertain due to variations in diagnostic criteria and the paucity of relevant literature. Several retrospective studies conducted in patients admitted to the emergency room have reported that the prevalence of hypertensive crisis among those presenting with HTN ranges from 16 to 54% (18, 19). In a survey conducted by the National Health and Nutrition Examination Survey in preadolescent and adolescent patients, the morbidity of hypertensive crisis was found to be between 1 and 4% (20). A single-center study in London, UK, reported that 24% of patients treated with HTN had severe HTN (5). Therefore, the incidence of pediatric HTN varies between studies due to the differences in study populations and inclusion criteria. In addition, the incidence of hypertensive emergency among children with hypertensive crisis varies from study to study. Our study found that the incidence of hypertensive emergency was approximately 65.71% in patients with hypertensive crisis. In a retrospective study from Taiwan, 84% of patients with hypertensive crisis had hypertensive urgency, while only 16% had hypertensive emergencies (21). However, in another recent study, 61% of pediatric patients with hypertensive crisis had a hypertensive emergency (22), which is similar to our study results.

In our study, the highest incidence of hypertensive crisis (54.29%) was observed in children aged 7–12 years, and the incidence was lowest in children aged <1 year. According to a study from Taiwan (4), 78% of patients diagnosed with hypertensive crisis were older than 7 years, and 44% were older than 13 years. However, in our study, only 17.14% of patients diagnosed with hypertensive crisis were older than 13 years. In addition, in the Taiwanese study, 12%−14% of patients who presented with hypertensive crisis were aged <1 year (4). In our study, the ratio of boys to girls with hypertensive crisis was 2:1; this was consistent with a previous study finding, which indicated a significant male predominance (4). In older patients with hypertensive crisis, an association with increased body mass index (BMI) has been reported (10, 23). However, only one patient in our study was obese, and no association was found between their BMI and hypertensive crisis.

A hypertensive crisis may be associated with any known cause of HTN. Unlike in adults, secondary HTN is the main cause of HTN in children (24), and this accounted for approximately 87.14% of all hypertensive crises in our study population. Among secondary factors, approximately half of the cases are due to renal disease, including renal artery stenosis, which was found in 44.12% of the patients. This percentage is much higher than that reported in a study conducted in Singapore (25). In our study, the second leading cause of hypertensive crisis was vascular disease, accounting for approximately 15.71% of cases. It is noteworthy that takayasu's arteritis is the predominant vascular disease, which has not been reported in other studies. In our study, approximately 52.63% of hypertensive crisis due to renal parenchymal disease were caused by end-stage renal disease, which is a higher percentage than that reported in studies from other countries (22, 25). A possible reason may be related to the fact that our hospital is the Children's Kidney Disease Center in South China, so we receive a larger number of referrals involving pediatric kidney disease. Our study also found the third major cause of hypertensive crisis was oncological disease and essential hypertension. The most common tumor was pheochromocytoma, followed by blastoma. The proportion of hypertensive crisis due to oncological disease was lower than that in Korea (22), but similar to that in Singapore (25). Essential HTN was also observed to be a cause of hypertensive crisis in this study, and mainly contributed to hypertensive urgency in adolescents, which was consistent with previous study findings (21). Subgroup analysis of children with hypertensive crisis found vascular diseases to be the main cause of both hypertensive emergency and urgency. In a study from Korea, cancer was found to be the main cause of hypertensive crisis in children, both in hypertensive emergency and hypertensive urgency (22). Another study from Singapore found that chronic kidney disease and obesity were the leading causes of hypertensive emergency, while cancer and obstructive sleep apnea were the leading causes of hypertensive urgency (25). These are the main differences observed in the etiology of hypertensive crisis between previous studies and our study. Therefore, physicians should be alert to the occurrence of a hypertensive crisis in children with vascular diseases, especially those with renal artery stenosis.

The clinical manifestations of hypertensive crisis in children are different from those of adults and may vary depending on the age and severity of end-organ damage (12, 22). Therefore, the early identification of hypertensive crisis in children is challenging, leading to frequent misdiagnoses. Our study showed that ≈44.29% of the patients, mainly school-aged children, complained of headaches. The other two most common clinical manifestations were visual symptoms and dizziness, which were also mainly seen in school-aged children. The proportion of convulsions was significantly higher in hypertensive emergency patients compared with hypertensive urgency patients, and the proportion of headaches was higher among those with hypertensive urgency than among those with hypertensive emergency. There were no significant differences in other symptoms between the two groups. These results suggest that common symptoms were not particularly closely associated with the severity of disease, but that severe manifestations such as convulsions should be considered a danger sign of hypertensive emergency. In addition, no significant differences were found in the incidence of dyslipidemia and increased creatinine between the two groups, which suggests that the occurrence of hypertensive crisis is most closely related to its etiology. Therefore, the identification of the etiological agent is the primary task in the assessment of hypertensive crisis.

End-organ damage is a serious complication of hypertensive crisis and a major risk factor for mortality. Common organ damage includes heart, retinal, kidney, and brain damage (12). In our study, retinal and brain damage were the most common types of organ damage, followed by heart damage. The clinical manifestations of retinal damage include decreased visual acuity, visual field defects, and retinal hemorrhage. In our study, almost all patients with visual symptoms were identified as having hypertensive emergencies, a finding corroborated by other study results (26–30). Of the known clinical presentations of hypertensive emergency, acute neurological signs are the most common and are a result of the disruption of the blood-brain barrier, insufficient oxygen delivery, and edema and microhemorrhages (29). Our study population included many patients with headache, dizziness, vomiting, and convulsions, which may be manifestations of acute cerebral parenchymal involvement. In addition, we found that ~73.91% of terminal organ damage in hypertensive emergency patients was single organ damage, and 26.09% was multiple organ damage. However, this finding has not been reported in other studies. Heart failure is a leading manifestation in hypertensive emergency patients, especially in infants (30, 31), and was consistently observed in our study findings. Identifying the key clinical features of hypertensive emergency is essential to adequately prepare for the effective evaluation of such patients. With the control of blood pressure, the acute injury of these end-organs also resolved, so the overall prognosis was relatively good.

The main purpose of hypertensive crisis treatment is to prevent or treat life-threatening complications caused by HTN (32, 33). Once a hypertensive crisis has been identified, immediate, safe, and effective intervention is required. In our study, most patients required a combination of multiple antihypertensive drugs. Although there are no uniform guidelines for the treatment of hypertensive crisis, intravenous antihypertensives are recommended in the initial treatment of hypertensive emergencies (7, 12). Due to different experiences, the proportion of intravenous antihypertensive used in clinical application is also different. In our study, 36.96% of patients with hypertensive emergency were treated with intravenous antihypertensives. This proportion was similar to that in Singapore (25) but significantly lower than that in Korean (22). With the accumulation of experience, we have used intravenous antihypertensives in all hypertensive emergency patients in the past three years. We recommend a gradual transition from intravenous to oral medication. Rapid conversion should be avoided, as it results in secondary injuries due to rebounding BP. In this study, there were no cases of mortality.

There are some limitations to our study. First, as hypertensive crisis is not common in children, the number of patients in our study was small. Second, this is a retrospective study, which is limited to inpatients and cannot reflect the characteristics of hypertensive crisis in the general pediatric population. Nevertheless, to our knowledge, this is the largest pediatric study of patients with hypertensive crisis and provides useful knowledge on the clinical features of hypertensive crises in children.

## Data Availability Statement

The original contributions presented in the study are included in the article/supplementary material, further inquiries can be directed to the corresponding authors.

## Ethics Statement

This study was approved by the Human Subjects Review Committee of the First Affiliated Hospital of Sun Yat-sen University. Written informed consent to participate in this study was provided by the participants' legal guardian/next of kin.

## Author Contributions

HB, YQ, and HW conceived and designed research. HB participated in all data collection and processing. HP and LX were the major contributors in organizing records and drafting the manuscript. All authors proof read and approved the manuscript.

## Funding

The study was supported by Guangdong Basic and Applied Basic Research Foundation (2020A1515010184).

## Conflict of Interest

The authors declare that the research was conducted in the absence of any commercial or financial relationships that could be construed as a potential conflict of interest.

## Publisher's Note

All claims expressed in this article are solely those of the authors and do not necessarily represent those of their affiliated organizations, or those of the publisher, the editors and the reviewers. Any product that may be evaluated in this article, or claim that may be made by its manufacturer, is not guaranteed or endorsed by the publisher.

## References

[1] Arbi G Pastor I Franco J. Diagnostic and therapeutic approach to the hypertensive crisis. Med Clin (Barc). (2018) 150:317–22. 10.1016/j.medcli.2017.09.027 29174704

[2] Nerenberg KA Zarnke KB Leung AA Dasgupta K Butalia S McBrien K. Hypertension Canada's 2018 guidelines for diagnosis, risk assessment, prevention, and treatment of hypertension in adults and children. Can J Cardiol. (2018) 34:506–25. 10.1016/j.cjca.2018.02.022 29731013

[3] Hansen ML Gunn PW Kaelber DC. Underdiagnosis of hypertension in children and adolescents. JAMA. (2007) 298:874–9. 10.1001/jama.298.8.874 17712071

[4] Wu HP Yang WC Wu YK Zhao LL Chen CY Fu YC. Clinical significance of blood pressure ratios in hypertensive crisis in children. Arch Dis Childhood. (2012) 97:200–5. 10.1136/archdischild-2011-300373 22241908

[5] Deal JE Barratt TM Dillon MJ. Management of hypertensive emergencies. Arch Dis Child. (1992) 67:1089–92. 10.1136/adc.67.9.1089 1417052 PMC1793638

[6] McNiece KL Poffenbarger TS Turner JL Franco KD Sorof JM Portman RJ. Prevalence of hypertension and prehypertension among adolescents. J Pediatr. (2007) 150:640–4. 10.1016/j.jpeds.2007.01.052 17517252

[7] Constantine E Linakis J. The assessment and management of hypertensive emergencies and urgencies in children. Pediatric Emerg Care. (2005) 21:391–6. 10.1097/01.pec.0000166733.08965.23 15942520

[8] Hari P Sinha A. Hypertensive emergencies in children. Indian J Pediatr. (2011) 78:569–75. 10.1007/s12098-010-0297-5 21271305

[9] Flynn JT Kaelber DC Baker-Smith CM Blowey D Carroll AE Daniels SR. Subcommittee on screening and management of high blood pressure in children. Clinical practice guideline for screening and management of high blood pressure in children and adolescents. Pediatrics. (2017) 140:e20171904. 10.1542/peds.2017-1904 28827377

[10] Joint Committee for Guideline Revision. 2018 Chinese guidelines for prevention and treatment of hypertension-a report of the revision committee of Chinese guidelines for prevention and treatment of hypertension. J Geriatr Cardiol. (2019) 16:182–241. 10.11909/j.issn.1671-5411.2019.03.014 31080465 PMC6500570

[11] Flynn JT. Evaluation and management of hypertension in childhood. Prog Pediatr Cardiol. (2001) 12:177–88. 10.1016/S1058-9813(00)00071-0 11223345

[12] Raina R Mahajan Z Sharma A Chakraborty R Mahajan S Sethi SK. Hypertensive crisis in pediatric patients: an overview. Front Pediatr. (2020) 8:588911. 10.3389/fped.2020.588911 33194923 PMC7606848

[13] Editorial Editorial Board of Chinese Journal of Pediatrics Subspecialty Subspecialty Group of Child Health Care The The Society of Pediatrics Chinese Medical Association Subspecialty Subspecialty Group of Cardiovascular Disease. Experts consensus for prevention and treatment of dyslipidemia in children and adolescents. Zhonghua Er Ke Za Zhi. (2009) 47:426–8. 10.3760/cma.j.issn.0578-1310.2009.06.007 19951468

[14] Li H Ji CY Zong XN Zhang YQ. Height and weight standardized growth charts for Chinese children and adolescents aged 0 to 18 years. Zhonghua Er Ke Za Zhi. (2009) 47:487–92. 10.3760/cma.j.issn.0578-1310.2009.07.003 19951507

[15] Yang Z Huang Y Qin Y Pang Y. Clinical characteristics and factors associated with hypertension in 205 hospitalized children: a single-center study in southwest *China Front Pediatr*. (2021) 9:620158. 10.3389/fped.2021.620158 33898356 PMC8058176

[16] Keith NM Wagener HP Barker NW. Some different types of essential hypertension: their course and prognosis. Am J Med Sci. (1974) 268:336– 45. 10.1097/00000441-197412000-00004 4616627

[17] Ross RD. The Ross classification for heart failure in children after 25 years: a review and an age-stratified revision. Pediatr Cardiol. (2012) 33:1295–300. 10.1007/s00246-012-0306-8 22476605

[18] Yang WC Wu HP. Clinical analysis of hypertension in children admitted to the emergency department. Pediatr Neonatol. (2010) 51:44–51. 10.1016/S1875-9572(10)60009-5 20225538

[19] Hari P Bagga A Srivastava N. Sustained hypertension in children. Indian Pediatr. (2000) 37:268–74. 10750068

[20] Din-Dzietham R Liu Y Bielo MV Shamsa F. High blood pressure trends in children and adolescents in national surveys, 1963 to 2002. Circulation. (2007) 116:1488–96. 10.1161/CIRCULATIONAHA.106.683243 17846287

[21] Yang WC Zhao LL Chen CY Wu YK Chang YJ Wu HP. First-attack pediatric hypertensive crisis presenting to the pediatric emergency department. BMC Pediatr. (2012) 12:200. 10.1186/1471-2431-12-200 23272766 PMC3538055

[22] Lee GH Lee IR Park SJ Kim JH Oh JY Shin JI. Hypertensive crisis in children: an experience in a single tertiary care center in Korea. Clin Hypertens. (2016) 22:10. 10.1186/s40885-06-0040-2 27092268 PMC4834822

[23] Reich A Muller G Gelbrich G Deutscher K Gödicke R Kiess W. Obesity and blood pressure: results from the examination of 2365 schoolchildren in Germany. Int J Obes Relat Metab Disord. (2003) 27:1459–64. 10.1038/sj.ijo.0802462 14634675

[24] Viera AJ Neutze D. Diagnosis of secondary hypertension: an age-based approach. Am Fam Phys. (2010) 82:1471–8. 21166367

[25] Lim AM Chong SL Ng YH Chan YH Lee JH. Epidemiology and management of children with hypertensive crisis: a single-center experience. J Pediatr Intensive Care. (2020) 9:45–50. 10.1055/s-0039-1698759 31984157 PMC6978164

[26] Saladini F Mancusi C Bertacchini F Spannella F Maloberti A Giavarini A. Diagnosis and treatment of hypertensive emergencies and urgencies among Italian emergency and intensive care departments. results from an Italian survey: progetto gear (gestione dell'emergenza e urgenza in area critica). Eur J Intern Med. (2020) 71:50–6. 10.1016/j.ejim.2019.10.004 31690479

[27] Vallelonga F Carbone F Benedetto F Airale L Totaro S Leone D. Accuracy of a symptom-based approach to identify hypertensive emergencies in the emergency department. J Clin Med. (2020) 9:2201. 10.3390/jcm9072201 32664670 PMC7408741

[28] Williams KM Shah AN Morrison D Sinha MD. Hypertensive retinopathy in severely hypertensive children: demographic, clinical, and ophthalmoscopic findings from a 30- year British cohort. J Pediatric Ophthalmol Strabismus. (2013) 50:222–8. 10.3928/01913913-20130319-01 23521027

[29] Ahn CH Han SA Kong YH Kim SJ. Clinical characteristics of hypertensive encephalopathy in pediatric patients. Korean J Pediatr. (2017) 60:266. 10.3345/kjp.2017.60.8.266 29042869 PMC5638725

[30] Seeman T Hamdani G Mitsnefes M. Share hypertensive crisis in children and adolescents. Pediatr Nephrol. (2019) 34:2523–7. 10.1007/s00467-018-4092-2 30276533

[31] Dionne JM Flynn JT. Management of severe hypertension in the newborn. Arch Dis Child. (2017) 102:1176–9. 10.1136/archdischild-2015-309740 28739634

[32] Nirali H Patel Sarah K Romero David C Kaelber. Evaluation and management of pediatric hypertensive crises: hypertensive urgency and hypertensive emergencies. Open Access Emerg Med. (2012) 4:85–92. 10.2147/OAEM.S32809.eCollection2012 27147865 PMC4753979

[33] Baker-Smith CM Flinn SK Flynn JT Kaelber DC Blowey D Carroll AE. Diagnosis, evaluation, and management of high blood pressure in children and adolescents. Pediatrics. (2018) 142:e20182096. 10.1542/peds.2018-2096 30126937

